# Editorial: Case reports in psychopharmacology, volume IV

**DOI:** 10.3389/fpsyt.2026.1861985

**Published:** 2026-05-12

**Authors:** Matej Stuhec, Patricia Di Ciano

**Affiliations:** 1 Faculty of Medicine, University of Maribor, Maribor, Slovenia; 2 Department of Clinical Pharmacy, Ormoz Psychiatric Hospital, Ormoz, Slovenia; 3 Institute for Mental Health Policy Research, Centre for Addiction and Mental Health, Toronto, ON, Canada; 4 Department of Pharmacology and Toxicology, University of Toronto, Toronto, ON, Canada; 5 Dalla Lana School of Public Health, University of Toronto, Toronto, ON, Canada; 6 Campbell Family Mental Health Research Institute, Toronto, ON, Canada

**Keywords:** adverse events, antipsychotics, psychiatric illness, psychopharmacology, treatment

This fourth volume in the case reports series focuses on psychiatric disorders. As with the previous three Research Topic (1–3), we highlight topics related to the treatment and management of these conditions. The contributions cover novel treatment strategies and draw our attention to potential new mechanisms of mental illness. Adverse events, such as tardive dyskinesia, are always a possibility, and this volume provides some advice on how to best use therapeutics, along with some cautionary notes. This Editorial presents novel approaches to the treatment of psychiatric conditions, followed by considerations for the use of these medications.

In this volume, some articles advance the treatment of psychiatric disorders with new approaches. Consistent with recent evidence suggesting that psychedelics could be effective in treating other psychiatric disorders (4, 5), Drange et al. demonstrated that psilocybin shows some, but not complete, promise in the treatment of obsessive-compulsive disorder (OCD) and related symptoms. In their study of twins discordant for OCD, the affected twin was administered psilocybin, which resulted in the remission of OCD symptoms. However, compared to the unaffected twin, cognitive deficits remained. Thus, psilocybin may be effective in treating certain aspects of OCD. In a different study, Ghaliani et al described the treatment of a case of post-traumatic confusional state (PTCS) in a 9-year-old boy with severe traumatic brain injury with paliperidone and adjunct risperidone. A number of agents, including risperidone, methylphenidate, olanzapine, and aripiprazole, were tried over a number of years, with no success. However, over two years after commencing treatment with long-acting injectable paliperidone palmitate with adjunct risperidone, the level of aggression markedly decreased.

This volume also presents some possible new etiologies of psychiatric disorders. Ozaki et al. highlighted the possibility that some psychiatric disorders may be caused by endocrine imbalances and respond better to endocrine treatment, such as in the case of a patient with psychiatric symptoms related to recurrent small-cell carcinoma of the cervix due to endogenous Cushing’s syndrome caused by ectopic adrenocorticotropic hormone production. In another report, Geiser et al. posited that vitamin C levels can affect the pharmacokinetics of lithium. In a patient with autism spectrum disorder and severe intellectual disability who presented with severe behavioral dysregulation, lithium levels failed to reach therapeutic levels. Following the diagnosis of vitamin C deficiency and the subsequent treatment of scurvy, serum lithium concentrations increased, and the patient showed rapid behavioral improvement.

This volume suggests that certain medications should be used with caution. Fesenmeier et al. described a case of venous thromboembolism (VTE) that emerged within hours of initiating treatment with olanzapine, quetiapine, levomepromazine, and lorazepam for treatment of a psychotic episode. This occurred earlier than the onset that is normally reported within the first few months or weeks of treatment and serves as a cautionary example of the use of antipsychotics. Furthermore, Qin et al. reported on selective serotonin reuptake inhibitor (SSRI)-associated vasculitis. In the patient reported, leukocytoclastic vasculitis (LCV) emerged 7 months after the initiation of fluoxetine therapy for generalized anxiety disorder. The symptoms resolved upon discontinuation of fluoxetine but recurred upon reinitiation of treatment with the same medication. Furthermore, subsequent treatment with paroxetine, another SSRI, resulted in a similar, albeit less severe, rash, suggesting cross-sensitization. Treatment with venlafaxine, a serotonin-norepinephrine reuptake inhibitor, did not result in the lesions. Finally, Fiala and Lenz reported on a menopausal woman who developed persistent genital arousal disorder after pharmacotherapy with trazodone, pregabalin, vaginal estriol, and depot medroxyprogesterone. The symptoms resolved upon complete discontinuation of all medications.

However, not all tales are cautionary: there is some new hope for psychiatric treatments. Preiss et al reported on the case of a patient who was exposed to milnacipran during pregnancy for the treatment of severe post-traumatic stress disorder and severe major depressive disorder with psychotic features. The woman had been taking milnacipran prior to pregnancy, but this was replaced with sertraline plus quetiapine due to the lack of safety data on milnacipran during pregnancy. Upon discontinuation of milnacipran, the symptoms returned, and, following a risk-benefit analysis, milnacipran was eventually resumed until gestational week 22, when it was discontinued due to moderate hyperemesis gravidarum. Three years after delivery, pediatric development screenings remained within normal limits. Similarly, Alshahrani et al. reported on three patients in whom tardive dyskinesia remitted after cessation of aripiprazole. This adds to the literature on the remitting nature of tardive dyskinesia.
